# *Campylobacter coli* From Retail Liver and Meat Products Is More Aerotolerant Than *Campylobacter jejuni*

**DOI:** 10.3389/fmicb.2018.02951

**Published:** 2018-12-12

**Authors:** Anand B. Karki, Daya Marasini, Clark K. Oakey, Kaitlin Mar, Mohamed K. Fakhr

**Affiliations:** Department of Biological Science, The University of Tulsa, Tulsa, OK, United States

**Keywords:** aerotolerance, hyper-aerotolerant, *Campylobacter*, retail liver, retail meat, oxidative stress, transcriptional regulators, catalase

## Abstract

Aerotolerance in the microaerophilic species *Campylobacter* was previously reported and could increase bacterial survival and transmission in foods during stressful processing and storage conditions. In this study, 167 *Campylobacter* isolates (76 *C. jejuni* and 91 *C. coli*) were screened for aerotolerance; these strains were previously isolated from retail chicken meat, chicken livers, chicken gizzards, turkey, pork, and beef liver samples. Bacterial cultures were incubated aerobically in Mueller Hinton broth with agitation and viable cell counts were taken at 0, 6, 12, and 24 h. Approximately 47% of the screened *Campylobacter* isolates were aerotolerant (viable after a 12-h aerobic incubation period), whereas 24% were hyper-aerotolerant (viable after a 24-h aerobic incubation). A greater prevalence of aerotolerant strains (80%) was found among *C. coli* isolates as compared to *C. jejuni* isolates (6%). Differences in the oxidative stress response related genes were detected among *C. jejuni* and *C. coli* isolates when comparative genomics was used to analyze 17 Whole Genome Sequenced (WGS) strains from our laboratory. Genes encoding putative transcriptional regulator proteins and a catalase-like heme binding protein were found in *C. coli* genomes, but were absent in the genomes of *C. jejuni*. PCR screening showed the presence of a catalase-like protein gene in 75% (68/91) of *C. coli* strains, which was absent in all tested *C. jejuni* strains. While about 79% (30/38) of the hyper-aerotolerant *C. coli* strains harbored the catalase-like protein gene, the gene was also present in a number of the aerosensitive strains. The Catalase like protein gene was found to be expressed in both aerobic and microaerobic conditions with a 2-fold higher gene expression detected in aerobic conditions for an aerosensitive strain. However, the exact function of the gene remains unclear and awaits further investigation. In conclusion, aerotolerant *Campylobacter* strains (especially *C. coli*) are prevalent in various retail meats. Further studies are needed to investigate whether the genes encoding catalase-like heme binding protein and putative transcriptional regulators in *C. coli* strains are involved in stress response.

## Introduction

Campylobacteriosis is a leading foodborne illness in developed countries, with symptoms including mild diarrhea and immunological disorders such as Guillian Barre syndrome (Dewey-Mattia et al., 2016). An increasing trend of *Campylobacter* infection has been reported in the USA from 2004 to 2012 at an annual rate of 11.4 cases per 100,000 individuals (Geissler et al., 2017). In 2014, 24 confirmed campylobacteriosis outbreaks with 324 confirmed illnesses were documented in the USA (Dewey-Mattia et al., 2016). *C. jejuni* accounts for more than 90% of clinical cases of campylobacteriosis, followed by *C. coli* with about 7% of clinical cases (Gillespie et al., 2002). *Campylobacter* is usually transmitted from poultry, but environmental sources also serve as transmission routes (Bronowski et al., 2014; Newell et al., 2017). Consumption of contaminated food products including retail meat, liver, dairy products, and water is commonly associated with clinical cases (Gillespie et al., 2002; Bronowski et al., 2014; Dewey-Mattia et al., 2016).

The prevalence of *Campylobacter* in retail meat and liver products has been reported (Noormohamed and Fakhr, 2012, 2013, 2014b; Huang et al., 2016). *C. jejuni* is predominant in retail meat products (mainly poultry products), whereas *C. coli* is prevalent in retail liver products and pork (Noormohamed and Fakhr, 2012, 2013, 2014b). *C. coli* strains from retail liver products were multidrug resistant and shared similar Sequence Type (ST) complexes with clinical isolates when subjected to Multilocus Sequence Typing (MLST) (Noormohamed and Fakhr, 2014a). The recent, increasing trend of antimicrobial resistance among *Campylobacter* strains indicates the potential threat of future outbreaks (Noormohamed and Fakhr, 2012, 2014b; Geissler et al., 2017).

*Campylobacter* is a microaerophilic, fastidious organism with an optimal growth temperature of approximately 42°C. Aerobic conditions, temperature variations, osmotic imbalances, and starvation are common stresses to *Campylobacter* during the processing and storage of retail meat and liver products (Bronowski et al., 2014; Bolton, 2015). The formation of viable but non-culturable (VBNC) state, biofilms, and aerotolerance are common strategies that enhance the viability of *Campylobacter* during stressful conditions (Bolton, 2015). Enhanced resistance to oxidative stress (Oh et al., 2015) and the production of oxidative stress response proteins (Rodrigues et al., 2016) are factors that likely increase the survival of *Campylobacter* exposed to aerobic conditions (Oh et al., 2015, 2017). A high incidence of aerotolerant *C. jejuni* from chicken was previously reported, with 35.7% of isolates identified as hyper-aerotolerant (HAT) (Oh et al., 2015). Furthermore, HAT strains had a higher prevalence of virulence genes than aerosensitive strains (Oh et al., 2017).

Most reports on the stress response of *Campylobacter* and gene expression analyses have been conducted with *C. jejuni* (Butcher et al., 2015; Handley et al., 2015). The availability of complete genome sequences for *C. coli* and *C. jejuni* from both retail meat and liver products (Marasini and Fakhr, 2016a,b,c, 2017a,b,c) has facilitated comparative genomic analyses. Furthermore, genomic differences in *C. coli* and *C. jejuni* strains (Fouts et al., 2005) might help to explain differences in aerotolerance (O'Kane and Connerton, 2017).

Previous reports from our laboratory showed high prevalence of *C. coli* and *C. jejuni* strains in retail liver products (Noormohamed and Fakhr, 2012, 2013, 2014b). Since the existence of aerotolerant strains would definitely enhance the survival of *Campylobacter* spp., we hypothesize that aerotolerant strains will be prevalent among those isolated from retail meats. The focus of the current study was to screen a large number of *C. jejuni* and *C. coli* strains from retail meat and liver products for aerotolerance. The presence of genes involved in the oxidative stress response were also explored among 17 *C. coli* and *C. jejuni* strains using sequence data previously generated in our laboratory.

## Materials and Methods

### Bacterial Strains and Growth Conditions

The *C*. *jejuni* (*n* = 76) and *C. coli* (*n* = 91) strains (Table S1) used in this study were previously isolated from retail chicken meat, chicken livers, chicken gizzards, turkey, pork, and beef livers (Noormohamed and Fakhr, 2012, 2013, 2014b). *Campylobacter* isolates were grown from stock cultures maintained at −70°C. Strains were inoculated to Mueller Hinton Agar (MHA) supplemented with 5% laked horse blood at 42°C for 48 h and incubated in microaerobic condition (6% O_2_, 13% CO_2_) in a water jacketed CO_2_ incubator (Thermo Scientific). Strains were transferred to fresh MHA with 5% laked horse blood and grown for 18 h prior to harvesting the cells for aerotolerance and hydrogen peroxide sensitivity assays.

### Screening for Aerotolerant *Campylobacter* Strains

Aerotolerance was assayed as described previously (Oh et al., 2015) with slight modifications. Briefly, *Campylobacter* cells were harvested after an 18-h incubation and adjusted to OD_600_ = 0.5 in PBS (pH = 7.4). OD_600_ = 1 was used for 51 samples to ensure bacterial inoculum >10^7^ CFU/ml). One ml of the *Campylobacter* cell suspension was then transferred to 9 ml of Mueller Hinton Broth (MHB) preincubated at 42°C in 50 ml Falcon tubes. Inoculated tubes with cracked open caps were incubated aerobically with agitation at 200 rpm in an incubator shaker with orbital diameter of 19 mm (New Brunswick I2400) at 42°C, and viable cell counts were obtained from 40 μl samples that were removed at 0, 6, 12, and 24 h. Aliquots (10 μl) from each dilution were spotted twice on MHA plates and incubated at 42°C for at least 48 h in microaerobic conditions (6% O_2_, 13% CO_2_). Each experiment was carried out in triplicate, and log CFU/ml values of viable cell counts were used for statistical analysis.

### Comparative Genomic Analysis

Comparative genomic analyses among *Campylobacter* isolates sequenced in our laboratory (Table 1; Marasini and Fakhr, 2016a,b,c, 2017a,b,c) and reference genome sequences (GenBank) were performed with Rapid Annotation using Subsystem Technology (RAST) (Aziz et al., 2008) and NCBI BLAST (https://blast.ncbi.nlm.nih.gov/Blast.cgi). Functional genomic comparisons were also performed for subsystems of the stress response and transcriptional regulators by RAST and BLAST. Multiple sequence alignment and phylogenetic analysis of nucleic acid sequences for catalase-like proteins from *Campylobacter* spp. from our study and GenBank (3/14/2017). (Table S2) were conducted using Geneious v. 11 (https://www.geneious.com).

**Table 1:** Whole genome sequenced (WGS) *Campylobacter* strains (from our laboratory) used for comparative genomic analyses.

***Campylobacter* strain**	**Source**	**Aerotolerance**	**Catalase- like protein**	**Accession number (chromosome and plasmids)**
*C. coli* HC2-48	Beef liver	Aerotolerant	–	CP013034.1, CP013035.1
*C. coli* CF2-75	Beef liver	Aerotolerant	–	CP013035.1, CP013036.1, CP013037.1
*C. coli* CO2-160	Beef liver	Aerotolerant	–	CP013032.1, CP013033.1
*C. jejuni* T1-21	Chicken meat	Sensitive	–	CP013116.1, CP013117.1
*C. jejuni* TS1-218	Chicken meat	Sensitive	–	CP017860.1, CP017861.1
*C. jejuni* FJ3-124	Chicken gizzard	Sensitive	–	CP017862.1
*C. jejuni* WP2-202	Chicken gizzard	Aerotolerant	–	CP014742.1, CP014743.1
*C. jejuni* ZP3-204	Chicken gizzard	Sensitive	–	CP017856.1, CP017854.1, CP017855.1
*C. coli* WA3-33	Chicken liver	Aerotolerant	+	CP017873.1, CP017874.1
*C. jejuni* OD2-67	Chicken liver	Sensitive	–	CP014744.1, CP014745.1, CP014746.1
*C. jejuni* IF1-100	Chicken liver	Sensitive	–	CP017863.1, CP017864.1
*C. coli* YF2-105	Chicken liver	Hyperaerotolerant	+	CP017865.1, CP017866.1, CP017867.1
*C. coli* BG2-108	Chicken liver	Hyperaerotolerant	+	CP017878.1, CP017879.1, CP017880.1
*C. coli* MG1-116	Chicken liver	Hyperaerotolerant	+	CP017868.1, CP017869.1, CP017870.1
*C. coli* BP3-183	Chicken liver	Hyperaerotolerant	+	CP017871.1, CP017872.1
*C. jejuni* YQ2-210	Turkey	Sensitive	–	CP017859.1, CP017857.1, CP017858.1
*C. coli* ZV1-224	Pork	Aerotolerant	–	CP017875.1, CP017876.1, CP017877.1

*Campylobacter sequences were previously described in Marasini and Fakhr (2016a,b,c); Marasini and Fakhr (2017a,b,c)*.

### Screening for Genes Encoding Catalase-Like Proteins

*Campylobacter* strains from stock cultures (−70°C) were inoculated to MHA supplemented with 5% laked horse blood and incubated for 48 h in microaerobic conditions (6% O_2_, 13% CO_2_) in a water jacketed CO_2_ incubator (Thermo Scientific) at 42°C. Strains were screened for genes encoding catalase-like heme-binding proteins by PCR using the following primers: forward, 5′-TCAACTCAATGCGGATCCTAAA-3′ and reverse, 5′-AGCATAAGCCTCGTTTCTTACA-3′.

PCR reactions were conducted as described previously (Noormohamed and Fakhr, 2012). Briefly, DNA samples were prepared in single cell lysis buffer. PCR reactions contained 3 μl of each DNA sample, 12.5 μl GoTaq Green master Mix (Promega, Madison, WI, USA), 7.5 μl water, and 1 μl of each forward and reverse primer. PCR cycle conditions included 95°C for 3 min, and 30 cycles of the following: 95°C for 1 min, 50°C for 1 min, 72°C for 1 min, followed by a final extension for 10 min at 72°C and hold at 4°C. PCR products were subsequently analyzed using agarose gel electrophoresis.

### Assay for Hydrogen Peroxide Sensitivity

Assay for hydrogen peroxide sensitivity was conducted as described previously with limited modifications (De Vries et al., 2017). Cell suspensions (OD_600_ = 0.15) were prepared in PBS (pH = 7.4) from 18 h cultures of 14 *Campylobacter* strains (nine *C. coli* and five *C. jejuni* isolates). Bacterial cell suspensions (4 ml) were mixed with 80 ml of MHA (~45°C), and 25 ml of each MHA-bacterial mixture was aliquoted into three, 90 mm petri dishes. Sterile filter paper discs (6 mm) containing 10 μl of H_2_O_2_ (0.05, 0.1, and 0.5 mM) were immediately placed onto the solid MHA-bacterial mixture, and inhibition zones were measured (*n* = 3) at 24 h.

### Isolation of Total RNA

Three *C. coli* strains containing catalase like gene (P1-18, WA3-33, MG1-116) were cultured in MHA supplemented with 5% laked horse blood with antibiotics (cefoperazone 20 μg/ml, vancomycin 20 μg/ml, trimethoprim 20 μg/ml, and amphotericin B 10 μg/ml) for 48 h in microaerobic condition (6% O_2_, 13% CO_2_) in a water jacketed CO_2_ incubator (Thermo Scientific) at 42°C. After 48 h incubation, bacterial suspension was adjusted to OD_600_ = 0.5 in MH broth and diluted 1::10 in MH broth. Bacterial inoculum (80 μl) was added to 20 ml freshly prepared MH broth and incubated at 42°C for 16 h (log phase) in microaerobic condition. Then, bacterial culture was divided equally and incubated under two conditions (microaerobic and aerobic incubation) for 1 more hour. For aerobic incubation, the bacterial broth was incubated aerobically in 25 ml conical flask with agitation at 200 rpm in an incubator shaker with orbital diameter of 19 mm (New Brunswick I2400) at 42°C. After 1 h incubation, bacterial broths in both aerobic and microaerobic conditions were subjected to RNA isolation. Each experiment was conducted in triplicates.

Multiple RNA samples were isolated for each strain and condition (microaerobic or aerobic) by using RNeasy Mini Kit (Qiagen) according to manufacturer's instruction. For total RNA isolation, 2 ml RNA protect bacteria reagent (Qiagen) was added to 1 ml of bacterial culture and vortexed for 5 s followed by incubation at room temperature for 5 min. Bacterial cell was harvested by centrifugation at 5,000 × g for 5 min at 4°C and cell pellet was resuspended in 700 μl of lysis buffer (RLT buffer, Qiagen) by vortexing for 10 s. Bacterial cell lysis was done by vortexing vigorously for 5 min in 2 ml safe lock tube containing ~30 mg acid washed glass beads (212–300 μm, Sigma, G1277). Lysate was centrifuged for 10 s at 13,800 × g (Heraeus Biofuge 13) and supernatant was transferred into a new tube. Equal volume of 70% ethanol was added to supernatant and mixed well by pipetting. Then, 700 μl of lysate was transferred to RNeasy spin column and centrifuged for 15 s at 13,800 × g. Flow through was discarded and remaining lysate solution was added and centrifuged in the same column. On-column DNA digestion with RNase Free DNase set (Qiagen) and RNA clean-up was done according to manufacturer's instructions. After washing and elution steps according to manufacturer's protocol, RNA samples were further subjected to DNA digestion in solution using RNase Free DNase set (Qiagen). DNA digestion process was carried out twice and cleaned up in new RNeasy spin columns. Quantity of total RNA was measured with NanoDrop™ 8,000 spectrophotometer. Absence of genomic DNA contamination in RNA samples was confirmed by PCR with primers of housekeeping genes *glyA* and *aspA*. All sequences of primers used in this study are listed in Table 2.

**Table 2:** List of Primers used in the qRT-PCR assay in this study.

**Primers**	**Sequences**	**Use**	**References**
*aspA*	5′-AGTACTAATGATGCTTATCC-3′ 5′-ATTTCATCAATTTGTTCTTTGC-3′	House keeping gene	(Noormohamed and Fakhr, 2014a)
*glyA*	5′-GAGTTAGAGCGTCAATGTGAAGG-3′ 5′-AAACCTCTGGCAGTAAGGGC-3′	House keeping gene	(Noormohamed and Fakhr, 2014a)
16SrRNA	5′-TGCTAGAAGTGGATTAGTGG-3′ 5′-GTATTAGCAGTCGTTTCCAA-3′	Enogenous control for qRT-PCR	(Koolman et al., 2016)
Catalase like protein gene	5′-TCAACTCAATGCGGATCCTAAA-3′ 5′-AGCATAAGCCTCGTTTCTTACA-3′	Target gene for qRT-PCR	This study

### qRT-PCR

The qRT-PCR assay was carried out in 96 well plates (MicroAmp Fast 96 well reaction plate, Applied Biosystem) using QuantiTect SYBR Green RT-PCR kit (Qiagen) according to the manufacturer's instructions. One step qRT-PCR was run in StepOne Real-Time PCR system (Applied Biosystems). 16S rRNA gene was used as reference gene for relative quantification of expressed catalase like gene in different treatments (aerobic vs. microaerobic). 16S rRNA gene had been used as reference genes for various studies (Klančnik et al., 2006; Koolman et al., 2016). Primers for target and endogenous reference used in qRT-PCR are listed in Table 2. Standard curve for both catalase like gene and 16S rRNA was created to determine efficiency of qRT-PCR using 10^−3^ to 10^−8^ dilution series of amplified PCR products from cDNA templates. For each RNA sample, four replicates were included in this study. In 25 μl reaction mixture, 12.5 μl QuantiTect SYBR Green RT-PCR Master Mix, 1 μl each of forward primer and reverse primer, 0.25 μl of QuantiTect RT mix, 8.25 μl RNase free water, and 2 μl RNA sample were included. Negative control for each sample without RT mix was included. One step qRT-PCR conditions included 50°C for 30 min (reverse transcription step), 95°C for 15 min, 40 cycles of the following steps: 94°C for 15 s, 50°C for 30 s, 72°C for 30 s (data collection step), that was followed by melting curve analysis step with 0.03°C/s temperature rise up to 95°C. Amplification efficiency of target gene and endogenous control was determined to be in range of 1.944 to 2. So, calculation of fold change in transcription level for each strain in aerobic vs. microaerobic condition was done using the Pffafl method (Pfaffl, 2004) using mean C_T_ values. Statistical analysis for relative comparison of transcription level was done using one-way ANOVA.

## Results

### Prevalence of Aerotolerant *C. coli* Strains

**Figure 1:**
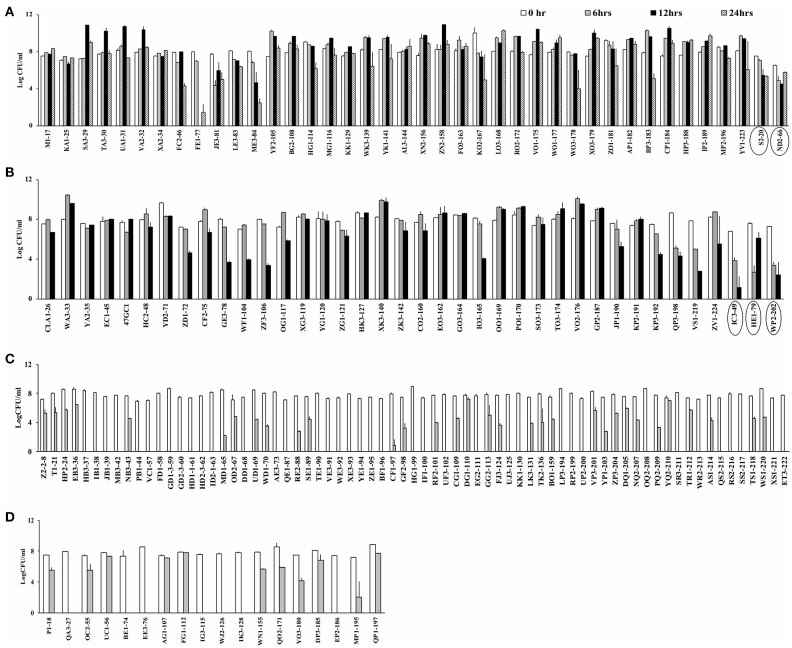
Screening for aerotolerant *Campylobacter* strains by incubating in MHB aerobically with agitation. **(A)** Hyper-aerotolerant *C. coli* and *C. jejuni* (circled) strains surviving a 24 h exposure to aerobic growth conditions. **(B)** Aerotolerant *C. coli* and *C. jejuni* (circled) strains surviving a 12 h exposure to aerobic conditions. Aerosensitive **(C)** *C. jejuni* and **(D)** *C. coli* strains not surviving the 12 h exposure to aerobic conditions. Error bars represent the mean standard error of triplicate experiments.

*Campylobacter* spp. (76 *C. jejuni* and 91 *C. coli* strains; Table S1) were screened for aerotolerance; 46.7% (78/167) were aerotolerant (viable after a 12-h incubation in aerobic conditions), whereas 23.9% (40/167) were hyper-aerotolerant (viable after a 24-h incubation in aerobic conditions) (Figure 1). Among the 76 *C. jejuni* strains, 6.6% (5/76) were aerotolerant and two strains from chicken meat and chicken liver were hyper-aerotolerant. A greater incidence of aerotolerant strains (80.2%, 73/91) was observed for *C. coli*; 100% of isolates from chicken gizzards (3/3), turkey (2/2) and pork samples (2/2), and 85.9% (49/57) of chicken liver isolates were aerotolerant. Similarly, 41.7% of all *C. coli* isolates were hyper-aerotolerant, and 49.1% (28/57) of chicken liver isolates could survive up to 24 h of aerobic incubation (Table 3).

**Table 3:** Screening *C. coli* and *C. jejuni* strains from retail meat and liver sources for aerotolerance.

***C. coli***	**Chicken**	**Chicken liver**	**Chicken gizzard**	**Beef liver**	**Turkey**	**Pork**	**Total**
Aerosensitive	3 (33.3%)	8 (14.1%)	–	7 (38.9%)	–	–	18 (19.8%)
Aerotolerant^*^	5 (55.5%)	21 (36.8%)	1 (33.3%)	5 (27.8%)	2(100%)	1 (50%)	35 (38.5%)
Hyperaerotolerant^**^	1 (11.1%)	28 (49.1%)	2 (66.6%)	6 (33.3%)	–	1 (50%)	38 (41.7%)
Total	9	57	3	18	2	2	91
***C. jejuni***							
Aerosensitive	21 (95%)	30 (96.8%)	7 (87.5%)	8 (80%)	5 (100%)	–	71 (93.4%)
Aerotolerant^*^	–	–	1 (12.5%)	2 (20%)	–	–	3 (3.9%)
Hyperaerotolerant^**^	1 (5%)	1 (3.2%)	–	–	–	–	2 (2.6%)
Total	22	31	8	10	5	–	76

* *Aerotolerant, viable after a 12 h incubation period in aerobic conditions*;

** *Hyperaerotolerant, viable after a 24 h incubation in aerobic conditions*.

### Oxidative Stress Subsystem and Transcriptional Regulators

A total of 17 WGS *Campylobacter* strains (8 *C. jejuni*, 9 *C. coli* strains) from our laboratory were used for comparative genomic analysis. Among WGS strains, only one *C. jejuni* WP2-202 but all WGS *C. coli* strains showed less sensitivity to aerobic conditions (Table 1). *C. coli* strains YF2-105, BG2-108, MG1-116, and BP3-183 from chicken liver were hyper-aerotolerant. Functional subsystem comparison of WGS *Campylobacter* strains (Table 1) by RAST and BLAST revealed relatively few genomic differences between *C. jejuni* and *C. coli* strains with respect to genes involved in oxidative stress (Table 4). Homologs for oxidative stress-related genes and transcriptional regulators were present in all sequenced *C. jejuni* and *C. coli* strains (Table 4). Transcriptional regulators for responsiveness to peroxide, e.g., RrpA (encoded by *cj1546*) and RrpB (*cj1556*), were previously shown to function in the aerobic stress response (Gundogdu et al., 2015); however, the sequenced *C. coli* strains in our study lacked homologs or orthologs for RrpA and RrpB (Table 4). Genes encoding the cytochrome c551 peroxidase precursor (*cj0020c* and *cj0358*) play a role in *Campylobacter* colonization (Bingham-Ramos and Hendrixson, 2008), and homologs for both genes were present in all WGS *C. jejuni* strains. Interestingly, *C. coli* strains lacked a homologs or orthologous sequence for *cj0020c*, which is involved in colonization (Bingham-Ramos and Hendrixson, 2008).

**Table 4:** Genes related to oxidative stress response in *Campylobacter*.

**Transcription regulators**	***Campylobacter jejuni*** **strains**									***Campylobacter coli*** **strains**								
	**NCTC11168**	**OD2-67**	**WP2-202**	**ZP3-204**	**IF1-100**	**YQ2-210**	**T1-21**	**FJ3-124**	**TS1-218**	**HC2-48**	**CF2-75**	**CO2-160**	**WA3-33**	**YF2-105**	**BG2-108**	**MG1-116**	**BP3-183**	**ZV1-224**
*Campylobacter* oxidative stress regulator (CosR)	*cj0355c*	99	99	99	99	99	99	99	99	97	97	97	98	98	98	97	99	98
Ferric uptake regulator (Fur)	*cj0400*	100	100	100	99	100	100	100	100	97	97	97	97	97	97	97	97	97
LysR-trpe regulator	*cj1000*	99	99	99	99	99	99	100	99	80^*^	80^*^	80^*^	80^*^	80^*^	80^*^	79^***^	80^*^	79^****^
Peroxide regulator (PerR)	*cj0322*	99	99	99	98	99	99	98	98	84	84	84	84	84	84	84	84	84
Putative Crp/Fnr family transcription regulator (BLD37_RS01065 in *C. coli* MG1-116)	–	–	–	–	–	–	–	–	–	100	100	100	100	100	100	100	100	100
Putative Peroxide stress regulator (Fur family) (BLD37_RS05205 in *C. coli* MG1-116)	–	–	–	–	–	–	–	–	–	99	99	99	100	100	100	100	100	99
Regulator of response to peroxide (RrpA)	*cj1556*	100	100	100 #	100	100 #	100	100 #	99 #	–	–	–	–	–	–	–	–	–
Regulator of response to peroxide (RrpB)	*cj1546*	100	100	100 #	100%	100 #	100	100 #	99 #	–	–	–	–	–	–	–	–	–
**OXIDATIVE STRESS RELATED GENES**																		
Alkyl hydroperoxide reductase (AhpC)	*cj0334*	100	100	100	100	100	100	100	100	97	97	97	97	97	97	97	97	97
Bacterioferritn comigratory protein (BCP)	*cj0271*	100	100	99	97	99	98	97	99	91^*^	91^*^	91^*^	91^*^	91^*^	91^*^	91^*^	91^*^	91^*^
Catalase (KatA)	*cj1385*	100	100	99	99	99	99	99	99	95^*^	95^*^	95^*^	95^*^	95^*^	95^*^	95^*^	95^*^	95^*^
Ankyrin repeat-containing putative periplasmic protein	*cj1386*	99	99	99	99	99	99	99	99	74	74	74	74	74	74	74	74	74
Catalase like heme binding protein (BLD37_01770 in *C. coli* MG1-116)	–	–	–	–	–	–	–	–	–	–	–	–	99	99	99	100	99	–
Desulforuberythrin (DRbr)	*cj0012c*	99	99	100	100	100	99	99	100	97	97	97	97	97	97	97	97	97
DNA-binding protein (Dps)	*cj1534c*	100	100	100	100	100	100	100	100	89	89	89	89	89	89	89	89	89
Methionine sulfoxide reductase (MsrA)	*cj0637c*	98	98	98^***^	96	98^***^	98	97	98	71^*^	71^*^	71^*^	71^*^	71^*^	71^*^	71^*^	71^*^	71^*^
Methionine sulfoxide reductase (MsrB)	*cj1112c*	100	100	99	99	99	99	99	98	91^**^	91^**^	91^**^	91^**^	91^**^	91^**^	91^**^	91^**^	91^**^
Superoxide dismutase (SodB)	*cj0169*	99	99	100	100	99	99	99	99	98	98	98	98	98	98	98	98	99
Cytochrome c551 peroxidase precursor (docA)	*cj0020c*	99	99	99	99	99	99	99	99	–	–	–	–	–	–	–	–	–
Cytochrome c551 peroxidase precursor	*cj0358*	99	99	99	99	99	99	99	99	92	92	92	92	92	92	92	92	92
Thiol peroxidase (Tpx)	*cj0779*	99^*^	99^*^	99^*^	99^*^	99^*^	99^*^	99^*^	99^*^	93^***^	93^***^	93^***^	93^***^	93^***^	93^***^	98^*^	93^***^	93^***^
Thiol-disulphide oxidoreductase [*trxC, C. jejuni* M1 (Acession: CP001900.1)]	*(cj1106) 98%*	98	98	98	98	98	98	98	99	83	83	83	83	83	83	83	83	83

*Percentages include sequence similarity of homologs and orthologs in C. jejuni and C. coli strains used in this study with protein sequences of the reference strain C. jejuni NCTC11168. Query cover was 100% unless stated separately*.

Query cover:

* * = 99%*,

** * = 97%*,

*** * = 96%*,

**** * = 90%, # = 86%*.

A putative transcriptional regulator sequence in the Crp/Fnr family (BLD37_RS01065, Table 5) was present in *C. coli* MG1-116, but was not identified in the WGS *C. jejuni* strains or the reference strain, *C. jejuni* NCTC11168. However, several *C. jejuni* strains associated with Guillian Barre syndrome (GenBank accession numbers: CP012689.1, CP012689.1, CP002029.1) and *C. jejuni* strains in the ST 677 clonal complex isolated from human feces and blood (Bioproject PRJNA268846) harbor orthologous sequences (~58% sequence similarity) of putative CRP/Fnr family transcriptional regulators. Similarly, another sequence for a putative peroxide stress regulator/ferric uptake regulation protein (Fur family) [(BLD37_RS05205) in *C. coli* MG1-116] (Table 5) was identified in all *C. coli* strains, but was absent in *C. jejuni* strains.

**Table 5:** Protein sequences in *C. coli* MG1-116 with putative roles in oxidative stress response.

**Sequence 1: Transcriptional regulator, Crp/Fnr family protein (BLD37_RS01065)** MDKEKILKEYFKNYNLENKDFEAMVEKSYFKEFDKNTILDDCLGFVIVLKGGF RAFILGQNAKEITVFKLKQNEECVICSHCIFETISYNLTLESFEDTQILIVPVKIYS QLKDKYPLIANYTLNLIAKRFNSLINILEQALFTPLHHRVKMFLKENAKEGKITF THEEIALHLGSTREVISRILKTMQKEGFIQQNRKEITLLKDL
**Sequence 2: Peroxide stress regulator / Ferric uptake regulation protein (Fur family) (BLD37_RS05205)** MEALELLKKHDIAITDLRVELLQILSLAKTPLSYDHFDIKANKTSFYRNMELFE KKGIVSKSELNRKSFYELADHAKAHFVCDKCHKISDVQMPKVKGTIKSVLIKGI CSDCEK
**Sequence 3: Catalase-like heme binding protein (BLD37_RS01770)** MKKYISSCLAICCLSSAIYANDVKYNAQKIADIFYQLNADPKNPKVKVNHAKG FCAMGTFEPAQSINKEIDVPLLTYKSLPIQVRYSLGGAFKDDKSKTRGMAIRIT DPQDSASWTMVMLNTEINFAKNPKEFGQFFEMRLPVNGKVDQEKISKMMQ EVDSYRNFAAYTDKIGISKSVANTPFFSIHTFYFKQADGENYLPARWKLVPSE GVAYLNEAQMKSASSDFLKEDFKDRVKTNKPVEYKMYLVYANKNDIINETTA LWSGKHKESLVGTFKVNALSDEDCNFDVYFPSDVPQGVNPPQDPLFDVRN EAYAITFSMRQ

### Catalase-Like Heme Binding Protein

**Figure 2:**
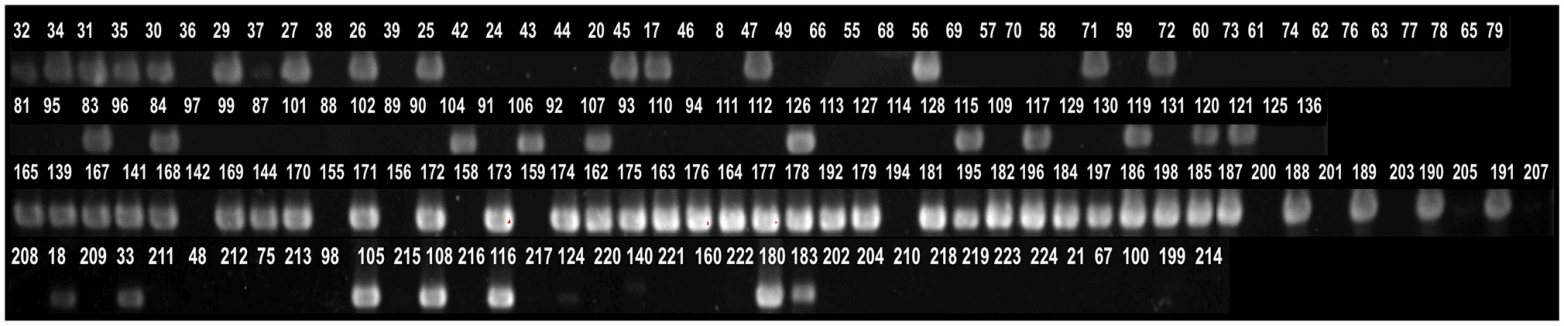
Detection of genes encoding a catalase-like heme binding protein by PCR. A 844-bp PCR product representing the gene was detected in many *Campylobacter* strains. The numbers above lanes represent the *Campylobacter* strain number (Table S1).

Comparative genomic analysis revealed that a gene encoding a catalase-like heme binding protein (BLD37_RS01770 in *C. coli* MG1-116, Table 5) was present in aerotolerant *C. coli* strains from chicken liver (e.g., strains WA3-33, YF2-105, BG2-108, MG1-116, and BP3-183; Table 4). However, WGS *C. coli* strains from other sources and all *C. jejuni* strains lacked the catalase-like gene sequence. We subsequently screened all 167 *Campylobacter* strains for the gene encoding the catalase-like heme binding protein by PCR analyses. PCR revealed an 844-bp product encoding the catalase-like protein in 74.7% (68/91) of all *C. coli* strains; this product was absent in 76 *C. jejuni* strains (Table 6, Figure 2). Most *C. coli* strains from poultry contained the gene encoding catalase-like heme-binding protein; these included strains from chicken meat (88.9%), chicken liver (94.7%), chicken gizzard (100%), and turkey, 100%. The incidence of the PCR product encoding the catalase-like gene was low to negligible in *C. coli* strains from beef liver (5.6%) and pork (0%) (Table 6).

**Table 6:** C. *coli* strains testing positive in PCR analysis for the gene encoding the catalase-like heme binding protein.

**Source**	**Aerosensitive**	**Aerotolerant**	**Hyperaerotolerant**	**Total**
Chicken meat	100% (3/3)	80% (4/5)	100% (1/1)	88.9% (8/9)
Chicken liver	100% (8/8)	90.5% (19/21)	96.4% (27/28)	94.7% (54/57)
Chicken gizzard	–	100% (1/1)	100% (2/2)	100% (3/3)
Beef liver	14.3% (1/7)	0/5	0/6	5.6% (1/18)
Turkey	–	100% (2/2)	–	100% (2/2)
Pork	–	0/1	0/1	0/2
Total	66.7% (12/18)	74.3% (26/35)	78.9% (30/38)	74.7% (68/91)

**Figure 3:**
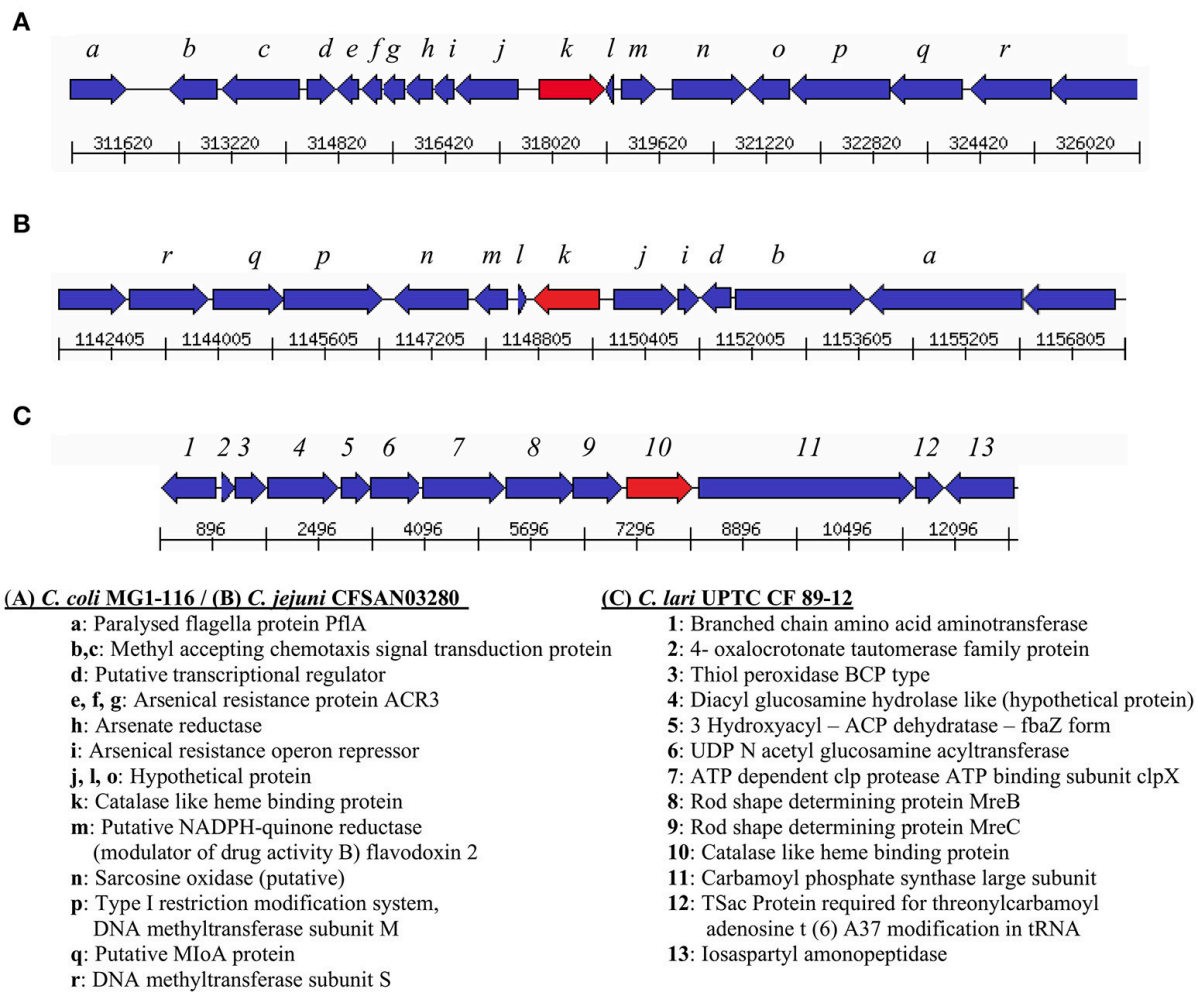
Arrangement of genes flanking the catalase-like heme binding protein (indicated by the red arrow) in **(A)** *C. coli* MG1-116 **(B)** *C. jejuni* CFSAN032806, and **(C)** *C. lari* UPTC CF 89-12.

**Figure 4:**
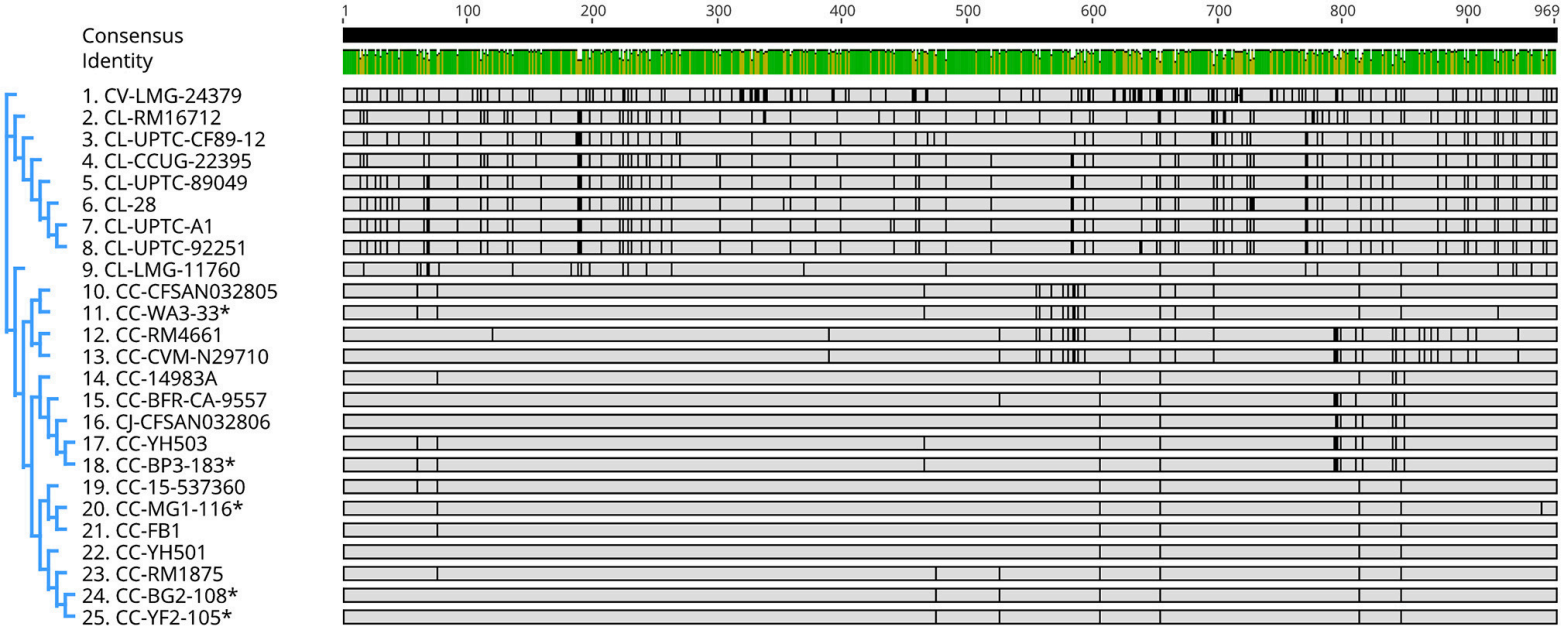
Nucleotide sequence similarity and phylogenetic analysis of genes encoding catalase-like heme binding proteins in *Campylobacter* (Table S2). Graphics were constructed using the neighbor-joining method (Tamura-Nei model) with Geneious V. 11. *C. coli* strains from the author's laboratory are marked with asterisks. Cc, *C. coli*; Cj, *C. jejuni*; Cl, *C. lari*; and Cv, *C. volucris*.

Genes encoding catalase-like heme-binding proteins were previously reported in urease-positive *C. lari* (Nakajima et al., 2016) and *C. jejuni* CFSAN032806 from chicken breast (GenBank accession no. CP023543.1). The genomic arrangement of the region containing the catalase-like gene in *C. coli* MG1-116 was compared with *C. jejuni* CFSAN032806 and *C. lari* UPTC CF 89-12 (Figure 3). The arsenate resistance operon was upstream of the catalase-like protein in *C. coli* MG1116 and divergently transcribed (Figure 3A, genes *e-i*). In *C. lari* UPTC CF 89-12, the catalase-like gene mapped adjacent to *mreB* and *mreC*, which are involved in determining morphological shape of the bacterial cell (Figure 3C, genes 8–9) (Nakajima et al., 2016). The gene encoding the catalase-like protein in *C. jejuni* CFSAN032806 is flanked by genes similar to those in *C. coli*, but lacks the genes encoding Acr3 and arsenate reductase (Figures 3A,B). Nucleotide similarity was highest between strains of the same *Campylobacter* species, and differences were greater between species (Table 7). Phylogenetic analyses indicated that the genes encoding the catalase-like proteins were more similar between *C. coli* and *C. jejuni*, which grouped in a different clade than *C. lari* and *C. volucris* (Figure 4).

**Table 7:**
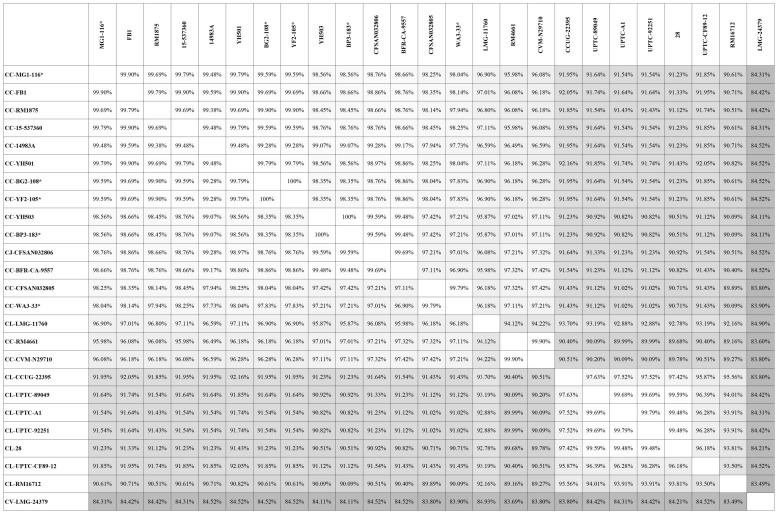
Nucleic acid sequence similarity of the gene encoding catalase-like heme binding protein in *Campylobacter* strains deposited in GenBank.

*Campylobacter strains from M. Fakhr laboratory; see Table S2 for accession numbers. CC, C. coli; CJ, C. jejuni; CL, C. lari, and CV, C. volucris). White rectangles indicate 100% identity; light gray shows lowest similarity.

**Figure 5:**
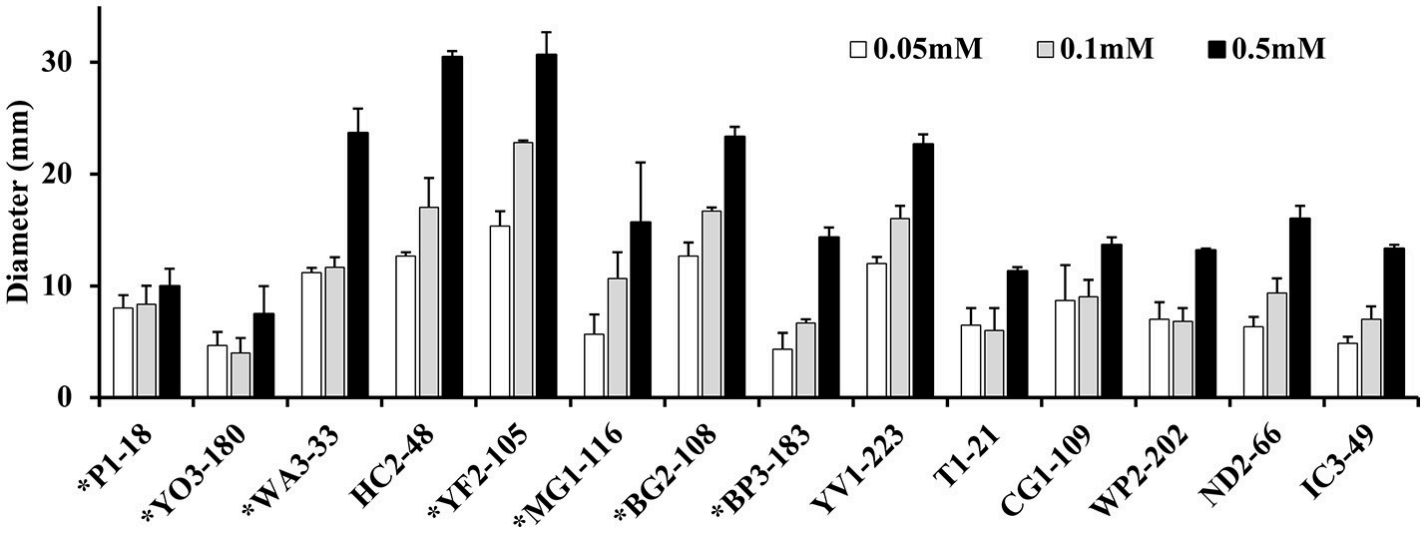
Assay for hydrogen peroxide sensitivity in *C. coli* strains P1-18 (CC18), YO3-180, WA3-33, YF2-105, MG1-116, BG2-108, BP3-183, HC2-48, and YV1-223 and *C. jejuni* strains T1-21, CG1-109, WP2-202, ND2-66, and IC3-49. Bacterial cells were exposed to different concentrations of H_2_O_2_ (0.05, 0.1, and 0.5 mM) and the diameter of diffusion zones was measured. *C. coli* strains with the gene encoding the catalase-like heme binding protein are indicated with asterisks. Vertical bars represent the standard error for the mean values from triplicate experiments.

**Figure 6:**
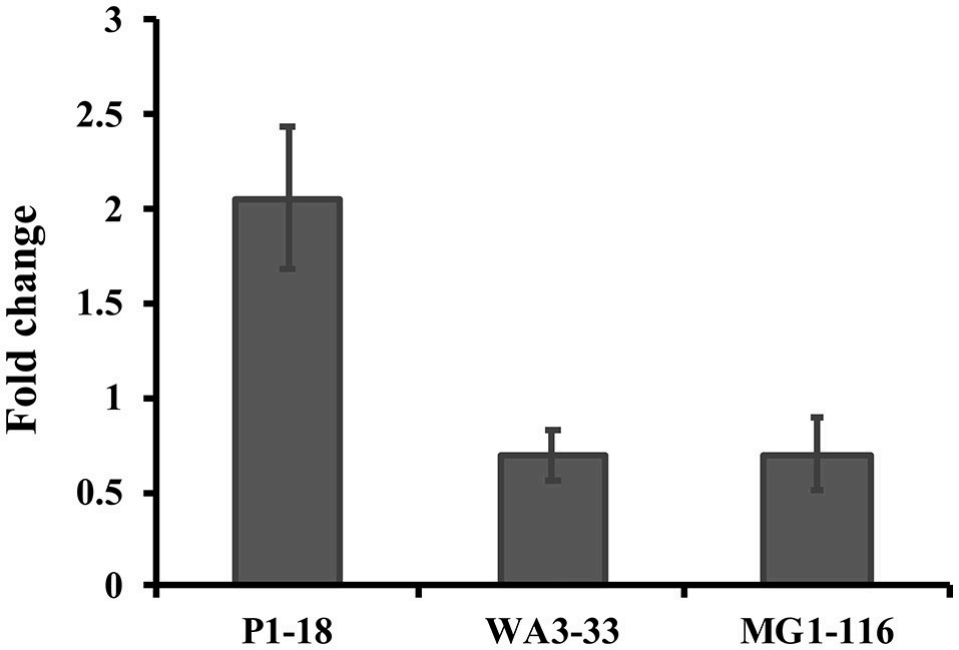
Relative quantification of catalase like protein gene expression in *C. coli* strains in aerobic condition compared to microaerobic condition. Hyperaerotolerant *C. coli* strain MG1-116, aerotolerant WA3-33, and aerosensitive P1-18 were used in this study.

Approximately 78.9% (30/38) of the hyper-aerotolerant, 74.3% (26/35) aerotolerant, and 66.7% (12/18) of aerosensitive *C. coli* strains contain the gene encoding the catalase-like heme binding protein (Table 6). Among 14 strains tested for hydrogen peroxide sensitivity, seven of *C. coli* strains harbored catalase like gene. Similarly, six of strains (including both *C. jejuni* and *C. coli*) were hyper-aerotolerant and four of them were aerotolerant. However, significant differences in H_2_O_2_ sensitivity among tested strains were neither correlated with aerotolerancy nor with the presence of the catalase-like gene (Figure 5). All three *C. coli* strains (P1-18, WA3-33, and MG1-116) used for gene expression study showed transcription of catalase like gene in both aerobic and microaerobic conditions. Hence, relative quantification of catalase like gene transcript level in aerobic condition vs. microaerobic condition was carried out for the three tested *C. coli* strains using 16S rRNA gene as endogenous control. No significant difference of transcript level was seen in aerobic vs. microaerobic condition for hyperaerotolerant *C. coli* strain MG1-116 and aerotolerant strain WA3-33 [Fold change: MG1-116 = 0.6973 (*p* > 0.05), WA3-33 = 0.696 (*p* > 0.05)] (Figure 6). Interestingly, the aerosensitive strain *C. coli* P1-18 had 2.0526-fold (*p* < 0.001) higher transcript level in aerobic condition when compared to microaerobic condition (Figure 6). However, in microaerobic condition, no significant difference of transcript level for the catalase like gene was seen between the hyperaerotolerant *C. coli* strain MG1-116, the aerotolerant strain WA3-33, and the aerosensitive strain P1-18 when normalized against the transcript level of the aerosensitive strain. In other words, the catalase like gene transcript level was relatively similar among the three strains when tested in microaerobic condition.

## Discussion

*Campylobacter* is an important foodborne pathogen that is transmitted from contaminated food products and water (Newell et al., 2017). Despite being microaerophilic and fastidious organism, many previous reports have reported the isolation of aerotolerant *Campylobacter* strains that could survive and grow in aerobic conditions (Rodrigues et al., 2015; Oh et al., 2017; O'Kane and Connerton, 2017). In our study, 46.7% (78/167) of tested *Campylobacter* strains were aerotolerant. Aerotolerant *Campylobacter* strains had shown prolonged survival during oxidative stress conditions (Oh et al., 2015, 2017; O'Kane and Connerton, 2017), increased biofilm formation (Bronnec et al., 2016b) and a high incidence of virulence genes (Oh et al., 2017) which help to survive in harsh environmental conditions. Even at low-temperature storage conditions, aerotolerant strains survive better than aerosensitive strains in different gaseous atmospheres (Oh et al., 2017). Hence, the high prevalence of *Campylobacter* reported in retail meat and liver products (Noormohamed and Fakhr, 2012, 2013, 2014b) seems to be associated with higher prevalence of aerotolerant *Campylobacter* strains found in this study. Our previous study reported the higher prevalence of *Campylobacter* in retail meat and liver products, where more than 75% of beef and chicken liver were contaminated with *C. coli* (Noormohamed and Fakhr, 2012, 2013). In the current study, 80.2% of the *C. coli* isolates from retail meat and liver products were aerotolerant; however, only 6.6% of *C. jejuni* were aerotolerant. The increased incidence of aerotolerant *C. coli* might be a contributing factor to the prevalence of *C. coli* in retail meat and liver products. Aerotolerant *C. jejuni* were not common in our study; however, Oh et al. found that 71% of *C. jejuni* strains from retail chicken were aerotolerant and 37% were hyper-aerotolerant (Oh et al., 2015). Although *C. coli* is the causal agent in only 7% of human clinical cases of campylobacteriosis (Gillespie et al., 2002), aerotolerant *C. coli* strains with a ST complex similar to clinical isolates (Noormohamed and Fakhr, 2014a) might result in additional clinical cases.

Various genes in *Campylobacter* function in the oxidative stress response (Flint et al., 2016), and most studies relevant to the oxidative stress response in *Campylobacter* have been conducted with *C. jejuni* (Butcher et al., 2015; Handley et al., 2015; Rodrigues et al., 2016). The oxidative stress response is modulated by many transcriptional regulators (PerR, Fur, RrpA, RrpB, CosR, CsrA) (Fields and Thompson, 2008; Hwang et al., 2011; Gundogdu et al., 2015; Handley et al., 2015; Flint et al., 2016), and some of these also function in iron transport (van Vliet et al., 2002; Holmes et al., 2005). Genes previously identified in the regulation of the oxidative stress response in *C. jejuni* (RrpA and RrpB) (Gundogdu et al., 2015, 2016) and colonization (*cj0020c*) (Bingham-Ramos and Hendrixson, 2008) were absent in the *C. coli* strains in our study. Mutagenesis study for MarR like transcriptional regulators RrpA and RrpB genes in *C. jejuni* strains have shown their role in oxidative and aerobic stress response. Mutants of these genes showed reduced survival under aerobic stress (Gundogdu et al., 2015, 2016). However, the absence of RrpA and RrpB seems to be distinctive genomic characteristics among *C. coli* strains (Gundogdu et al., 2016; O'Kane and Connerton, 2017). Comparative analysis of >4,000 *Campylobacter* genome sequences had shown the lack of RrpA and RrpB in *C. coli* strains (Gundogdu et al., 2016). A link between presence of transcriptional regulator like RrpB in *C. jejuni* strains with adaptation and survival capability of these strains in variable environmental conditions has been proposed (Gundogdu et al., 2016). Thus, it is likely that unique genomic traits and various putative transcription regulators found in *C. coli* strains might play a role in differential adaptation to oxidative stress. Presence of putative Crp/Fnr family transcriptional regulators in *C. coli* and some clinical *C. jejuni* strains might also indicate a possible association with virulence. The functional analyses of the transcriptional regulators identified in *C. coli* strains and their role in oxidative stress is underway in our laboratory.

In our study, comparative genomic analyses and PCR screening indicated that most *C. coli* isolates contained the gene encoding the catalase-like heme binding protein. The fact that none of the *C. jejuni* strains screened in our study possessed the gene is not surprising since out of all sequenced *C. jejuni* genomes in Genbank, only one *C. jejuni* CFSAN032806 genome harbors the catalase-like gene. Interestingly, *C. jejuni* CFSAN032806 and most of the *C. coli* isolates with the catalase-like protein gene originated from chicken or turkey, which might explain the greater prevalence of the catalase like protein gene in poultry isolates in the current study. A previously published report has shown presence of catalase-like gene in urease-positive *C. lari* strains (Nakajima et al., 2016). Sequence data from GenBank showed presence of catalase like gene in various species of *Campylobacter*. The high degree of sequence similarity in the catalase-like genes suggests the recent dissemination of this gene between strains; however, comparison of similar genes in *C. coli, C. jejuni*, and *C. lari* revealed inter-species variation in this region.

Although, a previous report (Oh et al., 2015) showed enhanced resistance toward hydrogen peroxide in aerotolerant strains, significant differences among hydrogen peroxide sensitivity could not be correlated among tested *Campylobacter* strains with their aerotolerancy in this study. Likewise, no significant difference in hydrogen peroxide sensitivity was observed between strains containing the catalase-like gene, and the gene was prevalent in both aerotolerant and aerosensitive strains. Nakajima et al. had also reported variable levels of catalase activity in *C. lari* strains that were independent of the presence of this gene (Nakajima et al., 2016). In a previous study, an aerotolerant *C. jejuni* strain showed higher transcript level for genes related to oxidative stress response in aerobic condition when compared to microaerobic condition (Rodrigues et al., 2016). A higher catalase equivalent activity in microaerobic condition was also reported in aerotolerant *C. jejuni* strains when compared to aerosensitive ones (Rodrigues et al., 2016). This was not the case in our study since the catalase like gene transcript level was relatively similar among the three hyperaerotolerant, aerotolerant, and the aerosensitive strains when tested in microaerobic condition. However, in the current study, higher expression of catalase like gene in aerobic condition was seen in aerosensitive *C. coli* strain P1-18 than aerotolerant strains (MG1-116 and WA3-33). Thus, it remains plausible that this gene may not be directly involved in conferring aerotolerancy but might help aerosensitive strains to slightly cope with oxidative stress. The absence of this gene in *C. jejuni* despite the presence of aerotolernat *C. jejuni* strains indicates that another mechanism might be involved in conferring aerotolerancy in *C. jejuni* and *C. coli* strains. The exact function of the gene encoding catalase-like heme binding protein remains unclear and awaits further investigation using mutagenesis and complementation.

Some prominent genomic differences were observed among the WGS strains, and these might contribute to discrepancies in aerotolerance as well as survival. A Type VI secretion system (T6SS) in *Campylobacter* had an enhanced hemolytic effect on blood cells and functioned in virulence (Lertpiriyapong et al., 2012; Bleumink-Pluym et al., 2013; Marasini, 2016). WGS strains *C. coli* ZV1-224 and *C. jejuni* (OD2-67, IF1-100, WP2-202, ZP3-204, YQ2-210, and TS1-218) harbor sequences for a T6SS (Marasini and Fakhr, 2016b, 2017a,b,c). A T6SS was also present in the previously reported aerotolerant *C. jejuni* Bf strain (Bronnec et al., 2016a,b) and *C. coli* OR12 strain (O'Kane and Connerton, 2017). In this study, *C. coli* ZV1-224 and *C. jejuni* WP2-202 were aerotolerant, but all other *Campylobacter* strains with putative T6SSs were aerosensitive. Hence, it is unlikely that the T6SS is a contributing factor for enhanced aerotolerance, a conclusion supported by a recent study (O'Kane and Connerton, 2017). The presence of a functional Entner Doudoroff (ED) pathway could enhance survival and biofilm formation in *Campylobacter* (Vegge et al., 2016). Thus, the ED pathway encoded by the *C. coli* ZV1-224 genome could contribute to enhanced aerotolerance, but further validation is needed.

O'Kane and Connerton recently demonstrated that relatively few genomic differences and mutations can create aerotolerance in a wild-type aerosensitive *Campylobacter* strain (O'Kane and Connerton, 2017). Hence, genomic differences between *Campylobacter* spp. (Fouts et al., 2005) might play a role in the differential aerotolerance. It is also possible that transcriptional and translational modifications might be sufficient to facilitate aerotolerance without significant genetic differences in genomic structure (Bronnec et al., 2016a,b).

In conclusion, aerotolerant *C. coli* strains are highly prevalent in retail meat and liver products. Aerotolerant *C. coli* strains with antimicrobial resistance and ST complexes similar to clinical strains pose a risk towards emerging clinical cases. Some genes encoding transcriptional regulators and a catalase-like protein are present in *C. coli* strains which are missing in *C. jejuni* strains. Although the catalase like gene is being transcribed in *C. coli* strains, its exact function in stress response or virulence is still not explored. Mutagenesis studies are currently underway in our laboratory to investigate the potential role of this gene in *C. coli*.

## Author Contributions

AK and MF research design and manuscript preparation. AK, DM, CO, and KM experimental procedures.

### Conflict of Interest Statement

The authors declare that the research was conducted in the absence of any commercial or financial relationships that could be construed as a potential conflict of interest.
