# Supplementary material for: Campylobacter coli From Retail Liver and Meat Products Is More Aerotolerant Than Campylobacter jejuni
Source: Front Microbiol. 2018 Dec 12;9:2951. doi: 10.3389/fmicb.2018.02951 (PMC6315125; doi:10.3389/fmicb.2018.02951)
Supplement: Supplementary file 1 [file Table_1.docx]

**SUPPLEMENTARY MATERIALS**

**TABLE S1** | Characteristics of *Campylobacter jejuni* (CJ) and *Campylobacter coli* (CC) strains in this study.

| **Strain** | **Source** | ***C. jejuni* / *C.coli*** | **Aerotolerance** | **Catalase-like protein** | **Accession number (chromosome and plasmids)** |
| --- | --- | --- | --- | --- | --- |
| HC2-48 | Beef liver | CC | Aerotolerant | - | CP013034.1, CP013035.1 |
| IC3-49 | Beef liver | CJ | Aerotolerant | - |  |
| OC2-55 | Beef liver | CC | Sensitive | - |  |
| AE3-73 | Beef liver | CJ | Sensitive | - |  |
| BE1-74 | Beef liver | CC | Sensitive | - |  |
| CF2-75 | Beef liver | CC | Aerotolerant | - | CP013035.1, CP013036.1, CP013037 |
| EF3-76 | Beef liver | CC | Sensitive | - |  |
| FE1-77 | Beef liver | CC | Hyper-aerotolerant | - |  |
| GE3-78 | Beef liver | CC | Aerotolerant | - |  |
| HE1-79 | Beef liver | CJ | Aerotolerant | - |  |
| JE3-81 | Beef liver | CC | Hyper-aerotolerant | - |  |
| CG1-109 | Beef liver | CJ | Sensitive | - |  |
| DG1-110 | Beef liver | CJ | Sensitive | - |  |
| EG2-111 | Beef liver | CJ | Sensitive | - |  |
| FG1-112 | Beef liver | CC | Sensitive | - |  |
| GG2-113 | Beef liver | CJ | Sensitive | - |  |
| HG1-114 | Beef liver | CC | Hyper-aerotolerant | - |  |
| IG3-115 | Beef liver | CC | Sensitive | + |  |
| HK3-127 | Beef liver | CC | Aerotolerant | - |  |
| IK3-128 | Beef liver | CC | Sensitive | - |  |
| JK1-129 | Beef liver | CC | Hyper-aerotolerant | - |  |
| KK1-130 | Beef liver | CJ | Sensitive | - |  |
| WN1-155 | Beef liver | CC | Sensitive | - |  |
| XN2-156 | Beef liver | CC | Hyper-aerotolerant | - |  |
| ZN2-158 | Beef liver | CC | Hyper-aerotolerant | - |  |
| BO1-159 | Beef liver | CJ | Sensitive | - |  |
| CO2-160 | Beef liver | CC | Aerotolerant | - | CP013032.1, CP013033.1 |
| ET3-222 | Beef liver | CJ | Sensitive | - |  |
| G2-2-8 | Chicken meat | CJ | Sensitive | - |  |
| P1-18 | Chicken meat | CC | Sensitive | + |  |
| S2-20 | Chicken meat | CJ | Hyper-aerotolerant | - |  |
| T1-21 | Chicken meat | CJ | Sensitive | - | CP013116.1, CP013117.1 |
| KA1-25 | Chicken meat | CC | Hyper-aerotolerant | + |  |
| LA1-26 | Chicken meat | CC | Aerotolerant | + |  |
| UC1-56 | Chicken meat | CC | Sensitive | + |  |
| VC1-57 | Chicken meat | CJ | Sensitive | - |  |
| GD1-3-59 | Chicken meat | CJ | Sensitive | - |  |
| GD2-3-60 | Chicken meat | CJ | Sensitive | - |  |
| HD1-1-61 | Chicken meat | CJ | Sensitive | - |  |
| HD2-3-62 | Chicken meat | CJ | Sensitive | - |  |
| ID2-1-63 | Chicken meat | CJ | Sensitive | - |  |
| UD1-69 | Chicken meat | CJ | Sensitive | - |  |
| XG1-119 | Chicken meat | CC | Aerotolerant | + |  |
| YG1-120 | Chicken meat | CC | Aerotolerant | + |  |
| ZG1-121 | Chicken meat | CC | Aerotolerant | + |  |
| UJ3-125 | Chicken meat | CJ | Sensitive | - |  |
| WJ2-126 | Chicken meat | CC | Sensitive | + |  |
| LK3-131 | Chicken meat | CJ | Sensitive | - |  |
| TK2-136 | Chicken meat | CJ | Sensitive | - |  |
| SR3-211 | Chicken meat | CJ | Sensitive | - |  |
| TR1-212 | Chicken meat | CJ | Sensitive | - |  |
| AS1-214 | Chicken meat | CJ | Sensitive | - |  |
| QS2-215 | Chicken meat | CJ | Sensitive | - |  |
| RS2-216 | Chicken meat | CJ | Sensitive | - |  |
| SS2-217 | Chicken meat | CJ | Sensitive | - |  |
| TS1-218 | Chicken meat | CJ | Sensitive | - | CP017860.1, CP017861.1 |
| VS1-219 | Chicken meat | CC | Aerotolerant | - |  |
| WS1-220 | Chicken meat | CJ | Sensitive | - |  |
| XS1-221 | Chicken meat | CJ | Sensitive | - |  |
| M1-17 | Chicken Gizzard | CC | Hyper-aerotolerant | + |  |
| HA2-24 | Chicken Gizzard | CJ | Sensitive | - |  |
| XA2-34 | Chicken Gizzard | CC | Hyper-aerotolerant | + |  |
| YA2-35 | Chicken Gizzard | CC | Aerotolerant | + |  |
| FD1-58 | Chicken Gizzard | CJ | Sensitive | - |  |
| FJ3-124 | Chicken Gizzard | CJ | Sensitive | - | CP017862.1 |
| UP2-200 | Chicken Gizzard | CJ | Sensitive | - |  |
| VP3-201 | Chicken Gizzard | CJ | Sensitive | - |  |
| WP2-202 | Chicken Gizzard | CJ | Aerotolerant | - | CP014742.1, CP014743.1 |
| YP1-203 | Chicken Gizzard | CJ | Sensitive | - |  |
| ZP3-204 | Chicken Gizzard | CJ | Sensitive | - | CP017856.1, CP017854.1,  CP017855.1 |
| QA3-27 | Chicken liver | CC | Sensitive | + |  |
| SA3-29 | Chicken liver | CC | Hyper-aerotolerant | + |  |
| TA3-30 | Chicken liver | CC | Hyper-aerotolerant | + |  |
| UA1-31 | Chicken liver | CC | Hyper-aerotolerant | + |  |
| VA2-32 | Chicken liver | CC | Hyper-aerotolerant | + |  |
| WA3-33 | Chicken liver | CC | Aerotolerant | + | CP017873.1, CP017874.1 |
| EB3-36 | Chicken liver | CJ | Sensitive | - |  |
| HB3-37 | Chicken liver | CJ | Sensitive | - |  |
| IB1-38 | Chicken liver | CJ | Sensitive | - |  |
| JB1-39 | Chicken liver | CJ | Sensitive | - |  |
| MB3-42 | Chicken liver | CJ | Sensitive | - |  |
| NB3-43 | Chicken liver | CJ | Sensitive | - |  |
| PB1-44 | Chicken liver | CJ | Sensitive | - |  |
| EC1-45 | Chicken liver | CC | Aerotolerant | + |  |
| FC2-46 | Chicken liver | CC | Hyper-aerotolerant | - |  |
| GC1-47 | Chicken liver | CC | Aerotolerant | + |  |
| MD1-65 | Chicken liver | CJ | Sensitive | - |  |
| ND2-66 | Chicken liver | CJ | Hyper-aerotolerant | - |  |
| OD2-67 | Chicken liver | CJ | Sensitive | - | CP014744.1, CP014745.1, CP014746.1 |
| PD1-68 | Chicken liver | CJ | Sensitive | - |  |
| LE3-83 | Chicken liver | CC | Hyper-aerotolerant | + |  |
| ME3-84 | Chicken liver | CC | Hyper-aerotolerant | + |  |
| QE1-87 | Chicken liver | CJ | Sensitive | - |  |
| RE2-88 | Chicken liver | CJ | Sensitive | - |  |
| SE1-89 | Chicken liver | CJ | Sensitive | - |  |
| TE1-90 | Chicken liver | CJ | Sensitive | - |  |
| VE3-91 | Chicken liver | CJ | Sensitive | - |  |
| WE3-92 | Chicken liver | CJ | Sensitive | - |  |
| XE3-93 | Chicken liver | CJ | Sensitive | - |  |
| YE1-94 | Chicken liver | CJ | Sensitive | - |  |
| ZE1-95 | Chicken liver | CJ | Sensitive | - |  |
| BF1-96 | Chicken liver | CJ | Sensitive | - |  |
| CF1-97 | Chicken liver | CJ | Sensitive | - |  |
| GF2-98 | Chicken liver | CJ | Sensitive | - |  |
| HF1-99 | Chicken liver | CJ | Sensitive | - |  |
| IF1-100 | Chicken liver | CJ | Sensitive | - | CP017863.1, CP017864.1 |
| RF2-101 | Chicken liver | CJ | Sensitive | - |  |
| UF3-102 | Chicken liver | CJ | Sensitive | - |  |
| WF1-104 | Chicken liver | CC | Aerotolerant | + |  |
| YF2-105 | Chicken liver | CC | Hyper-aerotolerant | + | CP017865.1, CP017866.1,  CP017867.1 |
| ZF3-106 | Chicken liver | CC | Aerotolerant | + |  |
| AG1-107 | Chicken liver | CC | Sensitive | + |  |
| BG2-108 | Chicken liver | CC | Hyper-aerotolerant | + | CP017878.1, CP017879.1, CP017880 |
| MG1-116 | Chicken liver | CC | Hyper-aerotolerant | + | CP017868.1, CP017869.1, CP017870.1 |
| OG1-117 | Chicken liver | CC | Aerotolerant | + |  |
| WK3-139 | Chicken liver | CC | Hyper-aerotolerant | + |  |
| XK3-140 | Chicken liver | CC | Aerotolerant | - |  |
| YK1-141 | Chicken liver | CC | Hyper-aerotolerant | + |  |
| ZK3-142 | Chicken liver | CC | Aerotolerant | - |  |
| AL3-144 | Chicken liver | CC | Hyper-aerotolerant | + |  |
| EO3-162 | Chicken liver | CC | Aerotolerant | + |  |
| FO3-163 | Chicken liver | CC | Hyper-aerotolerant | + |  |
| GO3-164 | Chicken liver | CC | Aerotolerant | + |  |
| IO3-165 | Chicken liver | CC | Aerotolerant | + |  |
| KO2-167 | Chicken liver | CC | Hyper-aerotolerant | + |  |
| LO3-168 | Chicken liver | CC | Hyper-aerotolerant | + |  |
| OO1-169 | Chicken liver | CC | Aerotolerant | + |  |
| PO1-170 | Chicken liver | CC | Aerotolerant | + |  |
| QO2-171 | Chicken liver | CC | Sensitive | + |  |
| RO2-172 | Chicken liver | CC | Hyper-aerotolerant | + |  |
| SO3-173 | Chicken liver | CC | Aerotolerant | + |  |
| TO3-174 | Chicken liver | CC | Aerotolerant | + |  |
| VO1-175 | Chicken liver | CC | Hyper-aerotolerant | + |  |
| VO2-176 | Chicken liver | CC | Aerotolerant | + |  |
| WO1-177 | Chicken liver | CC | Hyper-aerotolerant | + |  |
| WO3-178 | Chicken liver | CC | Hyper-aerotolerant | + |  |
| XO3-179 | Chicken liver | CC | Hyper-aerotolerant | + |  |
| YO3-180 | Chicken liver | CC | Sensitive | + |  |
| ZO1-181 | Chicken liver | CC | Hyper-aerotolerant | + |  |
| AP1-182 | Chicken liver | CC | Hyper-aerotolerant | + |  |
| BP3-183 | Chicken liver | CC | Hyper-aerotolerant | + | CP017871.1, CP017872.1 |
| CP1-184 | Chicken liver | CC | Hyper-aerotolerant | + |  |
| DP3-185 | Chicken liver | CC | Sensitive | + |  |
| EP2-186 | Chicken liver | CC | Sensitive | + |  |
| GP2-187 | Chicken liver | CC | Aerotolerant | + |  |
| HP3-188 | Chicken liver | CC | Hyper-aerotolerant | + |  |
| IP2-189 | Chicken liver | CC | Hyper-aerotolerant | + |  |
| JP1-190 | Chicken liver | CC | Aerotolerant | + |  |
| KP2-191 | Chicken liver | CC | Aerotolerant | + |  |
| KP3-192 | Chicken liver | CC | Aerotolerant | + |  |
| LP3-194 | Chicken liver | CJ | Sensitive | - |  |
| MP1-195 | Chicken liver | CC | Sensitive | + |  |
| MP2-196 | Chicken liver | CC | Hyper-aerotolerant | + |  |
| QP1-197 | Chicken liver | CC | Sensitive | + |  |
| QP3-198 | Chicken liver | CC | Aerotolerant | + |  |
| RP2-199 | Chicken liver | CJ | Sensitive | - |  |
| NQ2-207 | Chicken liver | CJ | Sensitive | - |  |
| OQ2-208 | Chicken liver | CJ | Sensitive | - |  |
| WD1-70 | Turkey | CJ | Sensitive | - |  |
| YD2-71 | Turkey | CC | Aerotolerant | + |  |
| ZD1-72 | Turkey | CC | Aerotolerant | + |  |
| DQ1-205 | Turkey | CJ | Sensitive | - |  |
| PQ2-209 | Turkey | CJ | Sensitive | - |  |
| YQ2-210 | Turkey | CJ | Sensitive | - | CP017859.1,  CP017857.1, CP017858.1 |
| WR2-213 | Turkey | CJ | Sensitive | - |  |
| YV1-223 | Pork | CC | Hyper-aerotolerant | - |  |
| ZV1-224 | Pork | CC | Aerotolerant | - | CP017875.1, CP017876.1, CP017877.1 |
