# Supplementary material for: Campylobacter coli From Retail Liver and Meat Products Is More Aerotolerant Than Campylobacter jejuni
Source: Front Microbiol. 2018 Dec 12;9:2951. doi: 10.3389/fmicb.2018.02951 (PMC6315125; doi:10.3389/fmicb.2018.02951)
Supplement: Supplementary file 2 [file Table_2.DOCX]

**SUPPLEMENTARY MATERIALS**

**TABLE S2** | All *Campylobacter* strains containing the gene for catalase-like heme binding protein available in GenBank.

***Campylobacter* strains Accession**

*C. coli* MG1116 * CP017868.1

*C. coli* FB1 CP011015.1

*C. coli* RM1875 CP007183.1

*C. coli* 15-537360 CP006702.1

*C. coli* 14983A CP017025.1

*C. coli* YH501 CP015528.1

*C. coli* YH503 CP025281.1

*C. coli* BG2108 * CP017878.1

*C. coli* YF2105 * CP017865.1

*C. coli* BP3183 * CP017871.1

*C. jejuni* CFSAN032806 CP023543.1

*C. coli* BFR-CA-9557 CP011777.1

*C. coli* CFSAN032805 CP023545.1

*C. coli* WA333 * CP017873.1

*C. lari* LMG 11760 CP007771.1

*C. coli* CVM N29710 CP004066.1

*C. coli* RM4661 CP007181.1

*C. lari* CCUG 22395 CP007776.2

*C. lari* UPTC CF89-12 AB736173.1

*C. lari* UPTC 89049 AB827928.1

*C. lari* UPTC 92251 AB827929.1

*C. lari* UPTC A1 AB827927.1

*C. lari* 28 AB827930.1

*C. lari* RM16712 CP007778.1

*C. volucris* LMG 24379 CP007774.1

**Campylobacter* strains from M. Fakhr laboratory.
